# Breast Lesions of Uncertain Malignant Potential (B3) and the Risk of Breast Cancer Development: A Long-Term Follow-Up Study

**DOI:** 10.3390/cancers15133521

**Published:** 2023-07-06

**Authors:** Chiara Bellini, Jacopo Nori Cucchiari, Federica Di Naro, Diego De Benedetto, Giulia Bicchierai, Andrea Franconeri, Irene Renda, Simonetta Bianchi, Tommaso Susini

**Affiliations:** 1 Diagnostic Senology Unit, Azienda Ospedaliero-Universitaria Careggi, 50134 Florence, Italy; bellinich@aou-careggi.toscana.it (C.B.); jakopo@tin.it (J.N.C.); dinarof@aou-careggi.toscana.it (F.D.N.); debenedettod@aou-careggi.toscana.it (D.D.B.); bicchieraig@aou-careggi.toscana.it (G.B.);; 2 Breast Unit, Gynecology Section, Department of Health Sciences, University of Florence, 50121 Florence, Italy; irene.renda@unifi.it; 3 Pathology Unit, Department of Health Sciences, University of Florence, 50121 Florence, Italy; simonetta.bianchi@unifi.it

**Keywords:** atypical ductal hyperplasia, atypical lobular hyperplasia, flat epithelial atypia, B3 lesions, breast cancer risk, surgical excision, follow-up mammography

## Abstract

**Simple Summary:**

The widespread use of screening mammography and breast ultrasound has enhanced early diagnosis and reduced breast cancer mortality. At the same time, this practice has led to an increase in breast biopsies for suspicious lesions, some of them ultimately resulting in breast lesions of uncertain malignant potential (B3 lesions). The correct management of B3 lesions is controversial, but surgical excision is generally recommended because of the considerable risk of an upgrade to cancer diagnosis upon final histology. Very little data exist concerning the role of B3 diagnosis as a risk factor for future development of breast cancer. The current study analyzes the largest series of B3 lesions from a single institution, providing new insights on both the risk of immediate upgrade to cancer and the subsequent risk of cancer development during the follow-up. An upgrade to carcinoma was found in 4.8% to 39.8% of B3 lesions depending on histologic subtype and in 22.7% on average. In the subsequent years, a diagnosis of breast carcinoma occurred in 9.2% of the patients. This information has considerable implications for future management of patients with B3 lesions.

**Abstract:**

Breast lesions of uncertain malignant potential (B3) are frequently diagnosed in the era of breast cancer (BC) screening and their management is controversial. They are generally removed surgically, but some international organizations and guidelines for breast research suggest follow-up care alone or, more recently, propose vacuum-assisted excision (VAE). The risk of upgrade to BC is known, but very little data exist on its role as risk factor for future BC development. We analyzed 966 B3 lesions diagnosed at our institution, 731 of which had long-term follow-up available. Surgical removal was performed in 91%, VAE in 3.8%, and follow-up in 5.2% of cases. The B3 lesions included flat epithelial atypia (FEA), atypical ductal hyperplasia (ADH), lobular intraepithelial neoplasia (LIN), atypical papillary lesions (PLs), radial scars (RSs), and others. Overall, immediate upgrade to BC (invasive or in situ) was 22.7%. After long-term follow-up, 9.2% of the patients were diagnosed with BC in the same or contralateral breast. The highest risk was associated with ADH diagnosis, with 39.8% of patients upgraded and 13.6% with a future BC diagnosis (*p* < 0.0001). These data support the idea that B3 lesions should be removed and provide evidence to suggest annual screening mammography for women after a B3 diagnosis because their BC risk is considerably increased.

## 1. Introduction

Breast cancer (BC) is the most frequent cancer among women with an increasing trend of incidence; however, mortality rates are slightly decreasing, even though it is still the leading cause of death among women due to oncologic pathology [1]. In recent years, the improvement in diagnostic techniques and screening campaigns have achieved the goal of early diagnosis of breast tumors in many instances. This, along with innovative surgical techniques and targeted biological treatments, has led to an increase in disease-free survival and overall survival [2]. Much evidence has shown how factors related to lifestyle and environment, such as physical activity, cigarette smoking, late pregnancy, and diet, play a pivotal role in primary prevention of breast cancer [3]. However, not all the factors related to the development of BC are modifiable with primary prevention practices. Non-modifiable risk factors include sex, age, genetic mutations, hormone-related factors, the density of breast tissue, a previous history of BC, and non-cancerous breast diseases like breast lesions of uncertain malignant potential (B3) [4].

B3 is a group of non-malignant breast lesions with a heterogeneous risk of concurrent or subsequent breast cancer diagnosis over time [5]. B3 lesions include atypical ductal hyperplasia (ADH), flat epithelial atypia (FEA), atypical lobular hyperplasia and classic-type lobular carcinoma in situ (LIN), papillary lesions (PLs), benign phyllodes tumours (PTs), radial scars (RSs), and other unclassified/not otherwise specified B3 lesions. They account for 5–10% of all breast biopsies [6], with an overall risk of malignancy which varies from 9.9 to 35.1% after open excision and large differences by histological subtype [7,8].

At present, the management of B3 lesions is still controversial since underestimation rates for percutaneous core needle biopsy for the B3 category are high overall [9], so it is important to find clinical, imaging, and histologic features that help us to rule out malignancy and tailor proper treatment for patients. Traditionally, B3 lesions have been treated with diagnostic surgical open excision (OE) because of the risk of upgrade to malignant lesions. However, since many of them were downgraded to benign lesions at final histology, in recent years more conservative approaches such as vacuum-assisted excision (VAE) and/or follow-up have been proposed [5,7,10]. Research efforts have focused on predictive factors of synchronous malignant lesions in the case of diagnosis of B3, and to our knowledge only one study evaluated their risk in terms of long-term follow-up [11]. Currently, there is no consensus nor guidelines from breast societies on the management and follow-up of women with surgically or percutaneously excised B3 lesions.

The aim of our study was to clarify the clinical meaning of breast lesions of uncertain malignant potential and to provide a contribution toward understanding the best approach to treatment and follow-up of these lesions. With this purpose, we assessed the risk associated with the diagnosis of a B3 lesion after core needle biopsy (CNB) or vacuum-assisted breast biopsy (VABB) in our monocentric experience in the last 20 years, both in terms of immediate upgrade to BC and future risk of BC development.

## 2. Materials and Methods

### 2.1. Patient Selection and Data Collection

From February 2001 to December 2019, we retrospectively reviewed CNBs and VABBs performed in our institution and included those with a histological diagnosis of B3. Out of a total of 29,696 breast biopsies, we identified 1182 patients with the diagnosis of a B3 lesion. We then excluded 216 patients who were referred to other institutions for surgical treatment or who had incomplete information, resulting in a final study population of 966 patients with B3 lesions.

Ultrasound (US)-guided CNBs were performed with a 14-gauge semi-automated biopsy gun (Precisa, Hospital Service, Rome, Italy), with a mean of 3 core samples obtained per lesion (range 3–6).

Tomosynthesis-guided VABBs were performed using an 11-gauge or 8-gauge needle (Mammotome revolve; Devicor Medical Products, Cincinnati, OH, USA) with the patients in a prone position (Affirm Prone Biopsy System; Hologic, Marlborough, MA, USA), obtaining a mean of 9 core samples per lesion (range 8–12).

A titanium marker clip (MammotomeMammomark 8G, DevicorMedical Products, Cincinnati, OH, USA) was positioned at the end of the VABB procedure to mark the site of the biopsy.

The histologic reports after biopsy with a diagnosis of a B3 lesion in our study included flat epithelial atypia (FEA), atypical ductal hyperplasia (ADH), atypical lobular hyperplasia (LIN1) and classic-type lobular carcinoma in situ (LIN2), that in the subsequent text will be collectively referred to as LIN, phyllodes tumors (PTs), papillary lesions (PLs), radial scars (RSs) and a group of rare histologic subtypes (mucocele-like lesions, myofibroblastoma, spindle cell proliferation) [12].

We retrospectively evaluated the mammograms and US exams performed at the time of the diagnosis and collected information on clinical suspicion and radiological suspicion according to the Breast Imaging Reporting and Data System (BIRADS) [13].

We also collected patient data from medical records, including the type of subsequent management after diagnosis (OE, VAE with post treatment histology, or follow-up).

We used final histology after surgery or VAE, when present, as the gold standard, considering as an upgrade the presence of invasive carcinoma or in situ carcinoma of the breast in the final specimens. We then evaluated the upgrade risk for each subgroup of B3 lesions.

After multidisciplinary team discussion for each of these patients, a mammogram and an ultrasound scan of the breast were performed every 12 months during the follow-up period. All the patients gave written informed consent for the use of their data for study purposes. All patient data were anonymized.

We retrospectively reviewed all follow-up exams, mammograms, and US imaging, and when present we reported the diagnosis of a subsequent breast cancer if histologically proven. We then analyzed the risk of developing a subsequent cancer in the homolateral or contralateral breast for each B3 lesion subtype.

Along with the occurrence of a diagnosis of breast cancer in the years of follow-up, for each histotype we evaluated the median age of patients, the initial median radiological suspicion according to BIRADS classification, the type of radiological lesion and its size, and the median follow-up time.

### 2.2. Statistical Analysis

Data analysis was performed using IBM SPSS Statistics, version 25.0. The frequency distribution was assessed using the chi-square test. The association between histologic diagnosis of B3 lesions, the degree of radiological suspicion, breast density, the type of treatment for the B3 lesion, and the risk of subsequent breast cancer diagnosis was investigated by multiple regression analysis. The interval between the diagnosis of each type of B3 lesion and the subsequent diagnosis of breast cancer was calculated according to the Kaplan–Meier method, and the differences were evaluated by the log-rank test.

## 3. Results

### 3.1. Patient Characteristics

The study included 966 patients with a histologic diagnosis of a B3 lesion after CNB or VABB, with a median age of 51.9 years (range 18–91) observed over a period of 19 years. Breast density, classified according to the BIRADS lexicon [13], was distributed as follows: 67 (6.9%) BIRADS A, 366 (37.9%) BIRADS B, 403 (41.7%) BIRADS C, and 130 (13.5%) BIRADS D. The most frequent localization of B3 lesions was the upper outer quadrant of the right breast.

Regarding the type of biopsy, 484 (50.1%) were US-guided CNBs while 482 (49.9%) were stereotactic or tomo-guided VABBs.

The distribution by histologic subtype of B3 lesions is indicated in Table 1.

Regarding management after biopsy, 879 (91%) B3 lesions underwent surgical OE, 37 (3.8%) VAE, and 50 (5.2%) follow-up. The type of management differed significantly by histotype (*p* = 0.004), as shown in Table 2.

### 3.2. Upgrade Rates

ADH was the histologic type with the highest risk of upgrade (39.8%), followed by papillary lesions (24.4%). Overall, the differences in upgrade rate by histotype were statistically significant (*p* < 0.001). Upgrade rates of B3 lesions to in situ carcinoma and to invasive carcinoma after open excision or VAE are shown in detail in Table 3.

### 3.3. Risk of Future Breast Cancer after B3 Diagnosis

Out of 966 women, we excluded those without follow-up information, leaving a total population of 731 women. Of these patients, we retrospectively reviewed the yearly mammography and US imaging performed in our institution and analyzed the risk of developing a future cancer during follow-up. Median follow-up time was 52.5 months (range 12–240 months).

**Figure 1:**
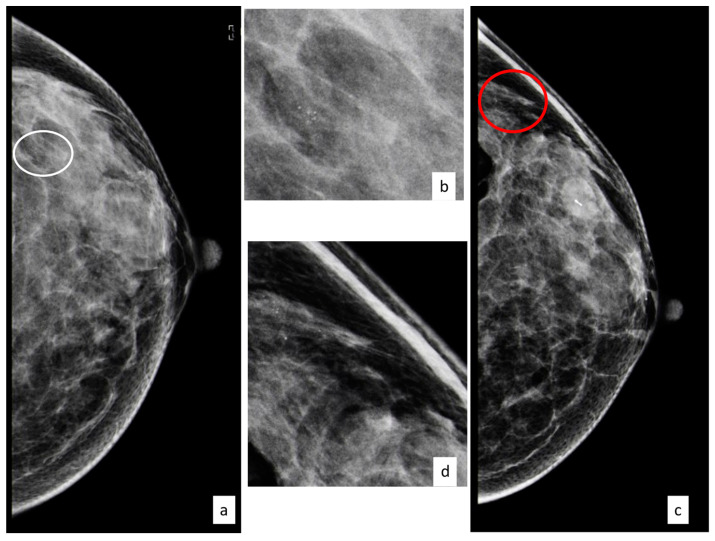
Forty-nine-year-old patient with a cluster of microcalcifications at the upper outer quadrant of the left breast ((**a**) CC left view, with the white circle showing microcalcifications; (**b**) magnifications); the woman underwent vacuum-assisted excision and after three years developed a new cluster of microcalcifications ((**c**) CC left view, with the red circle showing microcalcifications; (**d**) magnifications) that, after biopsy, turned out to be ductal carcinoma in situ.

Out of 731 women, a total of 67 (9.2%) developed a subsequent cancer in the homolateral or contralateral breast in the follow-up period. Figure 1 shows the clinical case of a patient with a diagnosis of a B3 lesion that developed a subsequent carcinoma in situ after 3 years.

**Figure 2:**
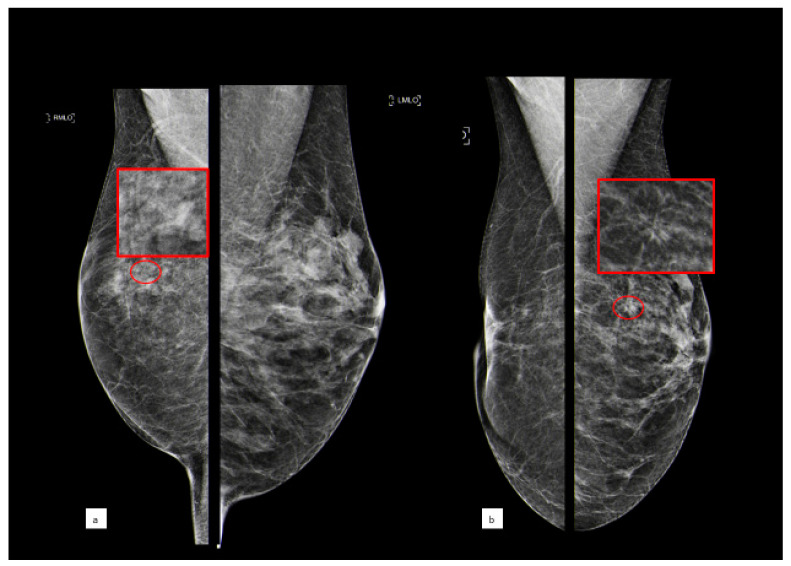
A 56-year-old patient with a cluster of microcalcifications at the upper outer quadrant of the right breast that was diagnosed as ADH after biopsy ((**a**) MLO bilateral view, with the red circle showing microcalcifications corresponding to a B3 lesion; the red square shows a magnification of this area); the woman underwent surgical excision and after five years developed an architectural distortion in the contralateral breast ((**b**) MLO bilateral view, with the red circle showing distortion; the red square shows a magnification of this area) that, after biopsy, turned out to be invasive ductal carcinoma.

Figure 2 shows the case of a B3 lesion in the right breast of a patient who developed an invasive breast cancer in the contralateral breast after 5 years.

No statistically significant difference (*p* = 0.814) was found in the risk of developing a subsequent cancer in terms of the type of management of the B3 lesion (OE, VAE, or follow-up), as shown in Table 4.

We then performed a linear regression analysis using future diagnosis of breast cancer as the dependent variable. Histology of the B3 lesions was the only significant independent predictor of future breast cancer development (*p* < 0.01), whereas the degree of radiological suspicion, breast density, and the type of treatment for the B3 lesions were not.

#### 3.3.1. Flat Epithelial Atypia

Of the 731 women, 112 had an initial diagnosis of FEA (15.3%), and the median age of this group was 48.7 years (range 33.5–80.0 years).

**Figure 3:**
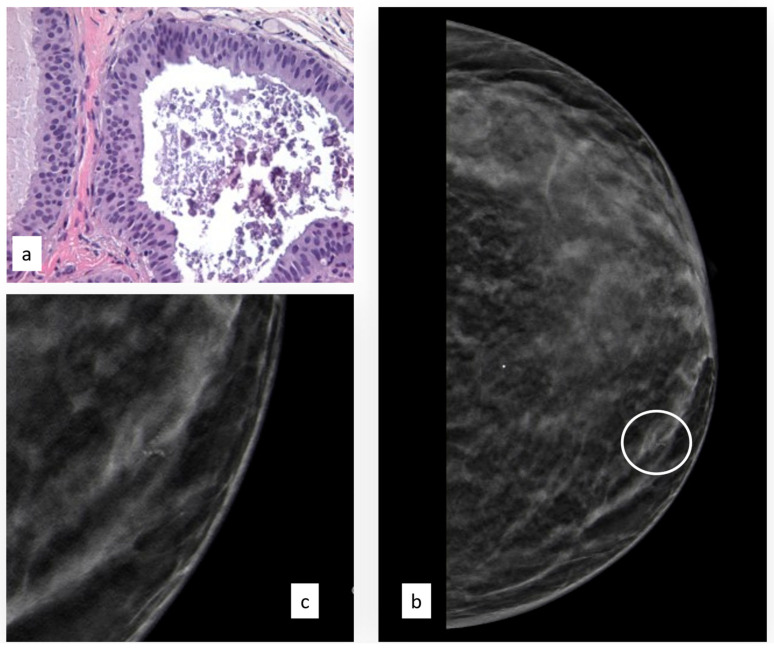
Histologic picture (**a**, Magnification (×20)) and radiological presentation ((**b**) left CC view; (**c**) magnification) of flat epithelial atypia (white circle).

The initial radiological findings were 82 microcalcifications, 28 masses, and two architectural distortions; the mean radiological suspicion level was BIRADS 3 (range 3–5). Illustrative radiologic and histologic examples of FEA are shown in Figure 3.

Of the 112 FEA patients, 8 (7.3%) developed subsequent breast cancer during follow-up, 2 (25%) in the same breast as the FEA and 6 (75%) in the contralateral breast.

The median time interval from first diagnosis of a B3 lesion to subsequent diagnosis of BC was 53.5 months.

#### 3.3.2. Atypical Ductal Hyperplasia

Of the 731 women, 200 had an initial diagnosis of ADH (27.4%), and the median age of this group was 54.5 years (range 17.5–86.0 years).

**Figure 4:**
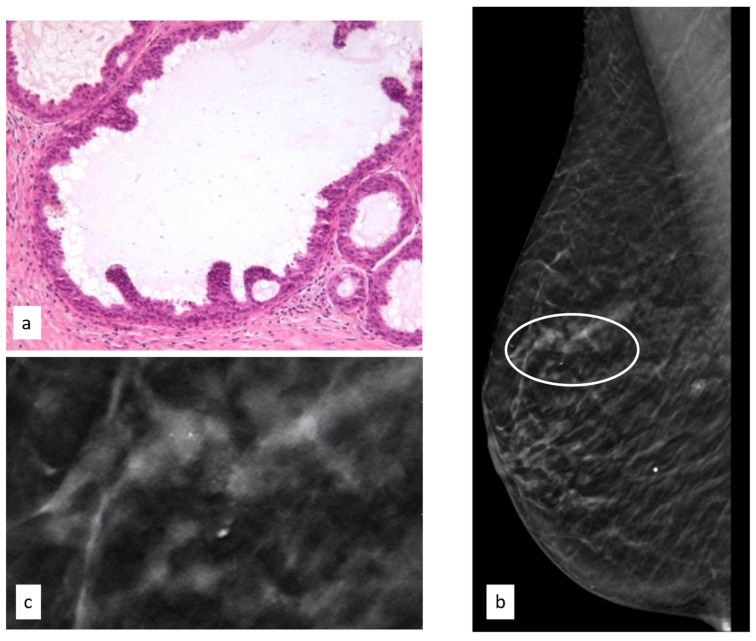
Histologic picture (**a**, Magnification (×20)) and radiological presentation ((**b**) right MLO view; (**c**) magnification) of atypical ductal hyperplasia (white circle).

The initial radiological findings were 114 microcalcifications, 84 masses, and two architectural distortions; the mean radiological suspicion level was BIRADS 4 (range 3–5). Illustrative radiologic and histologic examples of ADH are shown in Figure 4.

Of the 200 ADH patients, 27 (13.6%) developed subsequent breast cancer during follow-up, 18 (66.6%) in the same breast as the ADH and 9 (33.3%) in the contralateral breast.

The median time interval from first diagnosis of a B3 lesion to subsequent diagnosis of BC was 32 months.

#### 3.3.3. Lobular Intraepithelial Neoplasia

In total, 186 women had an initial diagnosis of LIN (25.4%), and the median age of this group was 52.7 years (range 33.7–83.5 years).

**Figure 5:**
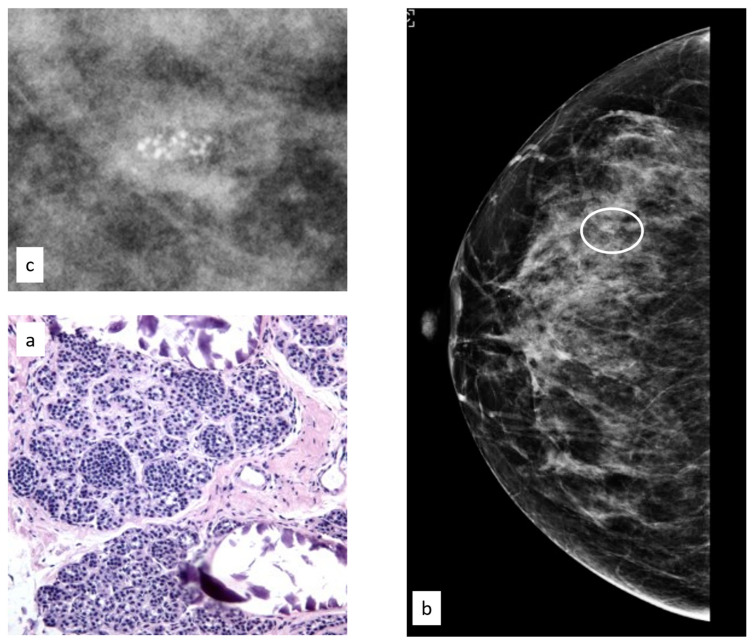
Histologic picture (**a**, Magnification (×20)) and radiological presentation ((**b**) right CC view; (**c**) magnification) of lobular intraepithelial neoplasia (white circle).

The initial radiological findings were 111 microcalcifications, 68 masses, and seven architectural distortions; the mean radiological suspicion level was BIRADS 4 (range 2–5). Illustrative radiologic and histologic examples of LIN are shown in Figure 5.

Of the 186 LIN patients, 17 (8.8%) developed subsequent breast cancer during follow-up, 11 (64.7%) in the same breast as the LIN and 6 (35.3%) in the contralateral breast.

The median time interval from first diagnosis of a B3 lesion to subsequent diagnosis of BC was 50.6 months.

#### 3.3.4. Atypical Papillary Lesion

In total, 93 women had the initial diagnosis of a PL (12.7%), and the median age of this group was 55.7 years (range 18.0–91.3 years).

**Figure 6:**
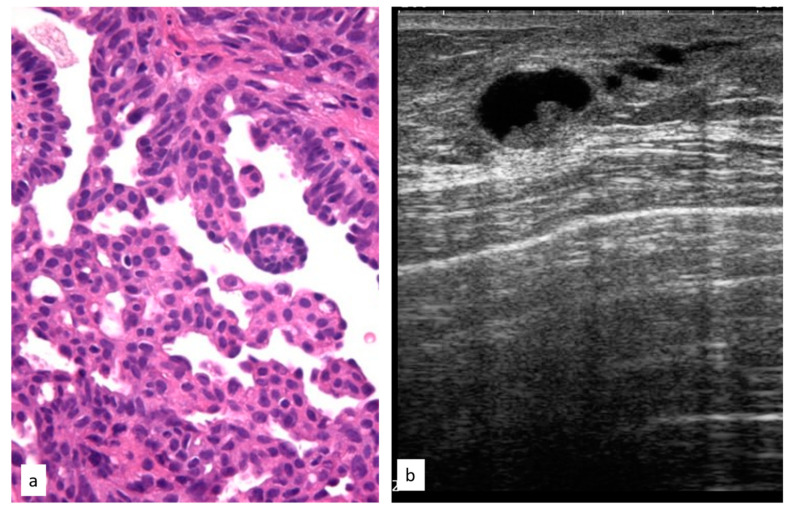
Histologic picture (**a**, Magnification (×20)) and ultrasound presentation (**b**) of atypical papillary lesions.

The initial radiological findings were two microcalcifications, 90 masses, and one architectural distortion; the mean radiological suspicion level was BIRADS 4 (range 2–5). Illustrative radiologic and histologic examples of atypical papillary lesions are shown in Figure 6.

Of the 93 PL patients, 9 (9.7%) developed subsequent breast cancer during follow-up after a median time interval of 51.1 months; in 7 (77.7%) cases the new cancer was in the same breast as the PL and in 2 (22.2%) cases the cancer was found in the contralateral breast.

#### 3.3.5. Phyllodes Tumors

In total, 36 women had the initial diagnosis of a PT (7.2%), and the median age of this group was 42.51 years (range 13.6–79.3 years).

**Figure 7:**
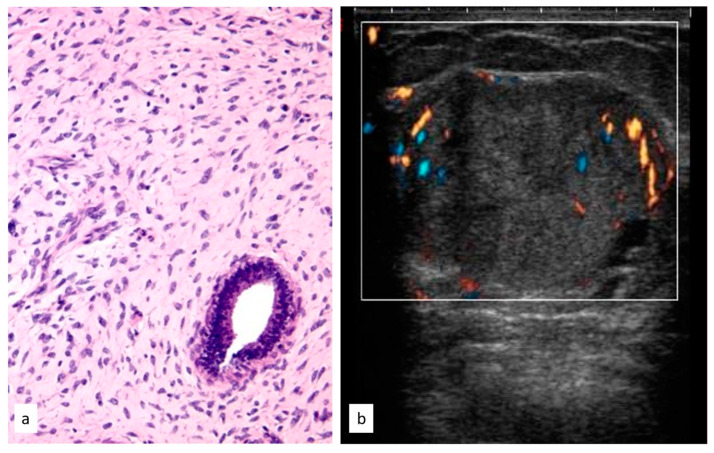
Histologic picture (**a**, Magnification (×20)) and ultrasound presentation (**b**) of phyllodes tumors.

All 36 PTs were reported as masses in mammograms and US images; the mean radiological suspicion level was BIRADS 3 (range 2–5). Illustrative radiologic and histologic examples of phyllodes tumors are shown in Figure 7.

Of the 36 PT patients, 2 (5.6%) developed a subsequent breast malignancy after a median time interval of 100.7 months; all of them were malignant phyllodes tumors, occurring in the same breast as the PL.

#### 3.3.6. Radial Scar

In total, 103 women had an initial diagnosis of RSs (14%), and the median age of this group was 47.9 years (range 24.9–81.7 years).

**Figure 8:**
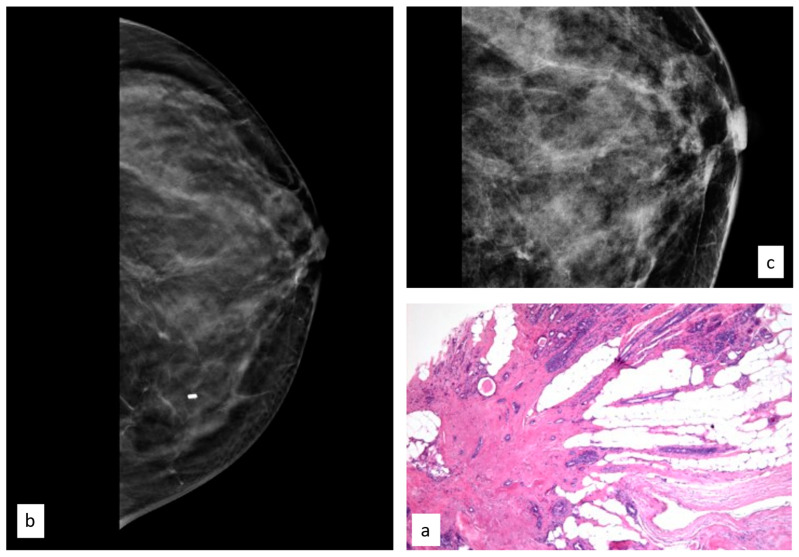
Histologic picture (**a**, Magnification (×10)) and radiological presentation ((**b**) right CC view; (**c**) magnification) of a radial scar (white circle).

The initial radiological findings were three microcalcifications, 58 masses, and 42 architectural distortions; the mean radiological suspicion level was BIRADS 4 (range 2–5). Illustrative radiologic and histologic examples of RSs are shown in Figure 8.

Of the 103 RS patients, 4 (3.9%) developed subsequent breast cancer during follow-up after a median time interval of 73.9 months. In three cases (75%) the cancer was found in the same breast as the RS, and in one case (25%) it was found in the contralateral breast.

**Figure 9:**
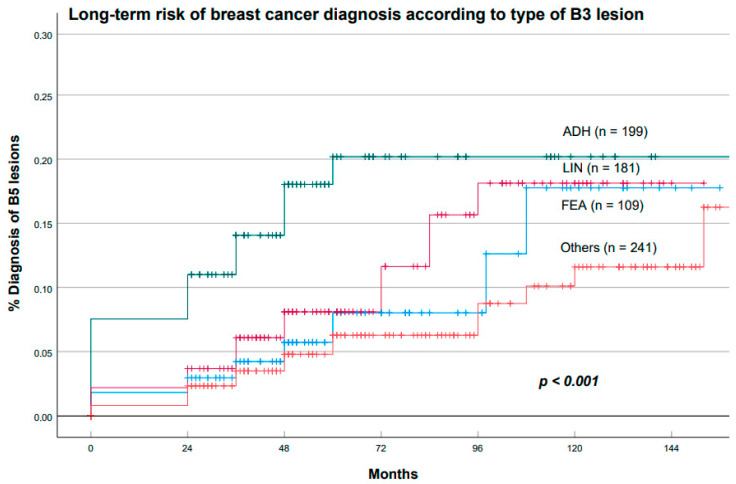
Cumulative incidence of BC during follow-up according to the histotype of the original B3 lesion. Abbreviations: atypical ductal hyperplasia (ADH); lobular intraepithelial neoplasia (LIN); flat epithelial atypia (FEA); ‘Others’ included papillary lesions, phyllodes tumors, radial scars, and rare histotypes.

We then performed a Kaplan–Meier estimation of the cumulative long-term risk of breast cancer associated with each type of B3 lesion. ADH was associated with the highest risk of BC diagnosis, followed by LIN and FEA. The differences were statistically significant (*p* < 0.001; log-rank test), as shown in Figure 9.

Overall, the mean interval between B3 diagnosis and breast cancer diagnosis in our sample was 38.0 months (range 2–180 months).

## 4. Discussion

In our series of 966 B3 lesions histologically diagnosed at our institution via CNBs or VABBs, we found an overall upgrade rate of 21.8% to BC (invasive or in situ) after OE or VAE, and this result is concordant with previous studies [6,12,13].

ADH histotype showed the highest upgrade risk (39.8%), followed by PLs (24.4%) and LIN (19.9%); the upgrade rates were concordant with data from the literature for ADH [14] and LIN [5,15,16], whereas the upgrade rate for PLs was slightly lower [17]. However, to our knowledge, there are no recent studies or meta-analyses on the upgrade rate of atypical papillary lesions, so we assume that this difference may be due to the better visualization of PLs thanks to improvements in US technology and better sampling with needles of larger caliper in more recent years [18].

The most important finding of our study was that the overall rate of developing subsequent BC during follow-up after diagnosis and excision of a B3 lesion was 9.2%. This result provides evidence that women with a B3 lesion diagnosis should be defined as “high-risk”. In fact, recent studies [14,19] evaluating age-specific 10-year absolute risk, with the goal of realizing risk-stratified breast cancer screening, indicated a threshold of 6% to define “high-risk” women. In particular, the three histotypes with higher risk of future cancer were ADH (13.6%), atypical PLs (9.7%), and LIN (8.8%); thus, in our opinion, these three categories could benefit from a tailored approach to surveillance and patients with these histotypes should not be discharged from clinical and radiological follow-up [20,21,22,23,24]. Recent guidelines published by a number of breast societies suggest that high-risk women should undergo an annual mammogram examination with an additional breast MRI, or breast US or contrast enhanced mammography in the case of contraindication to MRI or if MRI is not readily available [17,18,25]. Indeed, according to our results, women with B3 lesions should be advised to follow this pathway. The diagnosis of BC after initial diagnosis of a B3 lesion was made at a median time from follow-up of 38 months (range 2–180), so we suggest that at least a 5-year follow-up with annual mammogram and US would be a reasonable and cost-effective option for these patients.

To our knowledge, only one previous study analyzed the benefit of long-term clinical and radiological follow-up of surgically excised B3 lesions [11]. Hennessy et al. evaluated the risk of developing subsequent breast cancer in a 10-year retrospective series from a large tertiary symptomatic breast unit, analyzing 110 patients with surgically excised B3 lesions and reporting a 3.6% overall rate of future BC. In our series of 731 women with FU data available, the future risk of BC development was much higher. The higher incidence of BC during follow-up in the current series can be explained by a number of differences between our study and theirs. The study of Hennessy et al. was based on a much smaller sample with a shorter follow-up period and, above all, a different distribution of B3 subcategories. Hence, in their series, just 2.9% of surgically excised B3 lesions were of the ADH histotype, the category at higher risk for future BC development. In our study, ADH represented 27.4% of cases.

Our results are consistent with the study of Hartmann et al. [26], in which the authors examined the risk of developing future breast cancer in a large cohort of women who had previously received a histological diagnosis of a benign breast lesion after biopsy in a 15-year follow-up. They reported that after a biopsy with a diagnosis of a breast lesion with atypia, the risk of future BC was significantly increased, with a relative risk of 4.24.

It is known that after a diagnosis of ADH, and especially of LIN, the risk of developing BC is increased in both breasts and not just at the site of the B3 lesion [27]. This trend was also confirmed in the current series. However, an interesting finding in our study is that we found an increased risk of future breast cancer after a diagnosis of FEA, especially in the contralateral breast, with six of eight BC cases (75%) occurring in the contralateral side. We are not aware of previous reports of such a risk in association with the diagnosis of FEA. This result at least suggests that not only ADH, atypical PLs, and LIN, but also FEA represents a generic marker of BC risk that is not limited to the side of initial diagnosis.

Overall, in our study of 67 BC cases diagnosed during follow-up, 43 (64%) were homolateral to a prior B3 lesion, whereas 24 (36%) were contralateral; thus, our data support the role of uncertain malignant potential lesions of the breast as a risk factor for BC, irrespective of the side of the original lesion. Therefore, our results suggest that after diagnosis of a B3 lesion, bilateral radiological and clinical follow-up should be warranted.

In the current series, we found no significant differences in the future risk of developing breast cancer during follow-up independent of management type (OE, VAE, or follow-up). However, this result does not mean that surgical removal of B3 lesions may be omitted. In fact, patients submitted to OE were the large majority of our sample (91%), while just 5.2% underwent follow-up and 3.8% VAE; therefore, there is not enough statistical power to detect differences between the groups, as presented in Table 4. In addition, the absence of a statistically significant difference can be explained by the inhomogeneous distribution of lesions in the three groups: B3 lesions considered at higher risk like ADH, lesions > 15 mm in size, and those with discrepancy between radiological suspicion and histopathology were almost all sent to OE, whereas lesions considered at lower risk, smaller than <15 mm, and with low clinical suspicion sometimes led to follow-up. In the last years of the study, some of these low risk B3 lesions were treated with VAE, as recommended by recent European guidelines [5,28]. In addition, it has to be considered that, overall, 22.7% of patients with B3 lesions treated by OE or VAE were upgraded to BC, with upgrade risk being as high as 39.8% among ADH patients. Therefore, our study reinforces the idea that surgical removal of B3 lesions should always be considered, especially in the presence of ADH [29,30,31]. Other large-scale studies will be needed to investigate whether and when alternative management of B3 lesions may be feasible and whether the type of management may influence the future risk of BC diagnosis.

The strength of this study is that it is based on analysis of by far the largest series of B3 lesions ever published, while also including the longest follow-up period. This allowed us to accurately assess the risk of immediate upgrade to BC associated with the different subtypes of B3 lesions, as well as to shed light on the future risk of breast cancer diagnosis among women with a previous B3 lesion after core biopsy. Our study has some limitations. First, as stated above, there were discrepancies in the type of management after diagnosis. Second, the relatively extended period required for collection of data may have partially affected our results, since many improvements in sampling techniques and in diagnosis methods have taken place in recent years.

Nonetheless, we believe this is a real-world study that offers a robust contribution to understanding the role of B3 lesions as a risk factor for concurrent or future diagnosis of BC.

## 5. Conclusions

Diagnosis of a B3 lesion was associated with an overall rate of upgrade to BC of 22.7% after surgical removal. In addition, with a long-term follow-up, our study showed that after histological diagnosis of a B3 lesion, 9.2% of women subsequently developed breast cancer either homolaterally or contralaterally. The highest risk was associated with ADH diagnosis, with up to 39.8% of cases immediately upgraded to BC and 13.6% of women developing BC in subsequent years.

Our data support indications for the surgical removal of B3 lesions and provide evidence to suggest that a clinical and radiological follow-up with a yearly bilateral mammogram and US scan of the breast should be proposed for at least 5 years. This approach may possibly translate to earlier diagnosis of BC among patients with previous B3 lesion excision, with clear clinical benefits for these patients.

## Figures and Tables

**Table 1:** Patient distribution by histologic subtypes.

Histology	Nr. Patients	Percentage %
FEA	146	15.1
ADH	259	26.8
LIN	222	23
PL	124	12.8
PT	53	5.5
RS	145	15
Others/mucocele-like, myofibroblastoma, spindle cells proliferation	17	1.8
Total	966	100.0

Abbreviations: flat epithelial atypia (FEA); atypical ductal hyperplasia (ADH); lobular intraepithelial neoplasia (LIN); atypical papillary lesion (PL); phyllodes tumor (PT); radial scar (RS).

**Table 2:** Management of B3 lesions after biopsy according to different histotypes.

Histology	Open Excision	Vacuum-Assisted Excision	Follow-Up
FEA (*n* = 146)	123 (84.2%)	9 (6.2%)	14 (9.6%)
ADH (*n* = 259)	238 (91.9%)	6 (2.3%)	15 (5.8%)
LIN (*n* = 222)	207 (93.2%)	6 (2.7%)	9 (4.1%)
PL (*n* = 124)	115 (92.7%)	5 (4.0%)	4 (3.2%)
PT (*n* = 53)	53 (100%)	0 (-)	0 (-)
RS (*n* = 145)	128 (88.3%)	12 (8.3%)	5 (3.4%)
Others (*n* = 17)	15 (88.2%)	0 (-)	2 (11.8%)

Abbreviations: flat epithelial atypia (FEA); atypical ductal hyperplasia (ADH); lobular intraepithelial neoplasia (LN); atypical papillary lesion (PL); phyllodes tumor (PT); radial scar (RS). Others: mucocele-like, myofibroblastoma, spindle cells proliferation (*p* = 0.004).

**Table 3:** Overall upgrade rates according to different histotypes of the B3 lesions.

Histology	Upgrade to In Situ Carcinoma	Upgrade to Invasive Carcinoma	Total Upgrade Rate
FEA (*n* = 146)	3 (2.1%)	14 (9.6%)	17 (11.6%)
ADH (*n* = 259)	67 (25.9%)	36 (13.9%)	103 (39.8%)
LIN (*n* = 222)	18 (8.1%)	26 (11.8%)	44 (19.9%)
PL (*n* = 124)	9 (7.3%)	21 (17.1%)	30 (24.4%)
PT ^1^ (*n* = 53)	0 (-)	7 (13.2%)	7 (13.2%)
RS (*n* = 145)	5 (3.4%)	2 (1.4%)	7 (4.8%)
Others (*n* = 17)	1 (5.9%)	1 (5.9%)	2 (11.8%)
Total (*n* = 966)	103 (10.6%)	107 (11.1%)	210 (22.7%)

Abbreviations: flat epithelial atypia (FEA); atypical ductal hyperplasia (ADH); lobular intraepithelial neoplasia (LIN); atypical papillary lesion (PL); phyllodes tumor (PT); radial scar (RS). Others: mucocele-like, myofibroblastoma, spindle cells proliferation (*p* < 0.001). ^1^ From 53 phyllodes tumors classified as B3 lesions after core biopsy, 7 were upgraded to malignant phyllodes tumors after open excision; no upgrade to invasive ductal or lobular carcinoma was reported.

**Table 4:** Risk of future diagnosis of breast cancer according to the type of treatment for the B3 lesion.

Type of Treatment for the B3 Lesion	No Future Cancer during Follow-Up	Diagnosis of New Cancer during Follow-Up	Total
Open surgical excision	592 (90.7%)	61 (9.3%)	653
Vacuum-assisted excision	29 (90.6%)	3 (9.4%)	32
Follow-up	43 (93.5%)	3 (6.5%)	46

## Data Availability

The data presented in this study are available on request from the corresponding author.
